# The Efficiency of Energy Infrastructure Investment and Its Regional Economic Impact

**DOI:** 10.3390/ijerph20032125

**Published:** 2023-01-24

**Authors:** Lixin Kuang, Xiangrong Han, Guanyu Liu

**Affiliations:** 1 State Grid Jiangsu Electricity Co., Nanjing 210000, China; 2 School of Management and Engineering, Capital University of Economics and Business, Beijing 100070, China

**Keywords:** energy infrastructure, investment efficiency, regional economy, three-stage DEA

## Abstract

This study constructed an input–output and environmental indicator combination framework to evaluate the efficiency of energy infrastructure investment. Furthermore, the study used a three-stage DEA model to evaluate the efficiency of energy infrastructure investment projects in Jiangsu Province. Subsequently, the study constructed a system of indicators to measure regional economic development and assigns weights to them using the entropy value method, to obtain a comprehensive regional economic development score. Finally, this study analyzed the impact of energy investment efficiency on regional economic growth, economic stability, and industrial structure optimization. The study results show that the efficiency of energy infrastructure investment varies widely across Jiangsu and is highly correlated with the regional economic development model, the level of economic development, and the importance of the industry. The study also reveals that the improvement of energy infrastructure investment efficiency in Jiangsu fails to reflect the level of regional economic development; however, it has a crucial role in increasing social wealth and transforming the regional industrial structure and economy. Based on these results, this study further proposes countermeasures, such as planning a reasonable scale of investment, implementing differentiated regional investment, and upgrading management and technology.

## 1. Introduction

The construction of energy infrastructure is an important guarantee and power source for the further development of society and is particularly important for promoting economic development, advancing social progress, and enhancing national welfare. At the same time, enlarging energy infrastructure also brings environmental problems with its construction and may cause greater energy consumption in the future. Both of the above issues might bring new environmental challenges for the public administration. With continuous social and economic progress in recent years, China’s energy development has entered a new stage with new requirements. The new energy development strategy [1] has become the guide for developing the energy sector for the future and has opened up a new energy development model with unique characteristics.

At the same time, in the current process of social development, a series of environmental problems, such as the deteriorating climate and the increasing depletion of energy resources, need to be solved. Combining China’s primary national conditions and the current economic and social development stage, it is urgent to deepen the structural reform of energy infrastructure construction investment and improve the efficiency of energy and resource development and utilization efficiency. The Outline of the Fourteenth Five-Year Plan of the National Economic and Social Development of the People’s Republic of China and the Vision 2035 [2] state that it is necessary to coordinate and promote the construction of traditional infrastructure and new infrastructure, to build a modern infrastructure system that is complete, efficient, practical, intelligent, green, safe, and reliable. Therefore, adjusting to the new situation of energy development; building energy infrastructure that is clean, safe, and reliable; and promoting the improvement of investment efficiency have become the basic requirements for its construction [3].

As the core of the energy industry and the basis for the energy market, the demand for construction investment in electric power infrastructure in China is expanding. As the most essential part of energy infrastructure, electric power infrastructure is of great significance to guaranteeing the daily life of residents, meeting the needs of industrial production, and promoting the efficiency of energy resource allocation. The investment in power infrastructure construction is characterized by significant demands and complex investment types, but the current investment in power infrastructure suffers from a low effective utilization of resources, unreasonable allocation of invested resources, and unbalanced regional development. The difference in the efficiency of energy infrastructure investment in different regions is relatively significant; and within the energy industry, the problem of an uneven efficiency of the different construction enterprises and construction projects prevails, and there is significant room for improvement. Combined with the national requirements for coordinated regional development, investment control, and supply-side energy reform, it is particularly important to study the efficiency of infrastructure investment and improve the input–output efficiency of construction enterprises and construction projects, which is important for the sustainable and healthy development of China’s economy and environment.

As one of the important energy infrastructure investment, electricity infrastructure investment is closely related to national development, social progress, and residents’ quality of life, and helps to improve the utilization rate of energy resources and their safety. However, electricity infrastructure investment projects generally have a long construction period, large scale of capital investment, and large impact on society. Moreover, to improve the efficiency of electricity power infrastructure investment, administrations must consider the cost and gains of construction from a practical point of view. In 2020, the National Development and Reform Commission of China promulgated the “Provincial Power Grid Transmission and Distribution Price Pricing Measures”, which states that the profitability model should be based on the approved transmission and distribution price revenue [4]. Therefore, it is necessary to consider each construction project comprehensively; reduce unnecessary capital investment; as well as adjust unreasonable cost inputs, control project input costs, and improve the efficiency of investment.

This study took electricity power infrastructure investment in Jiangsu as an example, and the analysis established an investment efficiency evaluation index system that is scientific and feasible. In addition, this study used a three-stage DEA to evaluate its efficiency scientifically and rationally. Furthermore, the efficiency values derived from the empirical analysis were utilized to further study the impact of energy infrastructure investment efficiency on regional economic growth, stability, and optimization of industrial structure.

## 2. Literature Review

Production efficiency is usually defined as the pareto optimum achieved by economic activities of a specific scope and industry type under specific resource allocation conditions. The efficiency of production, with the maximization of economic activity as the core measure, is of particular interest to experts, because it is a good indicator of the management quality and level of production. Farrell’s research, in 1957, used the production frontier function to study the input–output efficiency of capital and human resources, i.e., productivity, and put forward the concept of efficiency evaluation for the first time [5]. However, this approach does not allow for the analysis and study of multiple output indicators. On this basis, Afriat [6] proposed establishing a production frontier function to evaluate the efficiency of decision units using parametric efficiency. Charnes, Cooper, and Rhodes proposed the first non-parametric technical efficiency analysis method, i.e., the DEA research method, in 1978, and created the CCR model to achieve efficiency measurement under a constant scale payoff. The obtained efficiency includes the component of scale efficiency and is generally referred to as the integrated technical efficiency value [7]. However, in practical applications, a large proportion of evaluation units are not in the best input state and scale to meet production efficiency requirements. To address the efficiency error due to the assumption of constant returns to scale, in 1984, Banker, Charnes, and Cooper proposed a DEA model for efficiency evaluation with variable returns to scale, to estimate improve the scale efficiency of decision units [8], i.e., the BCC model. In 2002, Fried showed that the efficiency values derived from DEA model analysis are influenced by internal management factors, external environmental factors, and random disturbance term factors and could not accurately evaluate input–output efficiency under the influence of internal management factors only. After further research, Fried [9] refined and extended the traditional DEA model using the SFA model proposed by Aigner [10] et al. and Meeusen [11] et al. and proposed a three-stage DEA model, which was analyzed in three stages, and the final input–output efficiency value obtained was the efficiency after excluding the influencing factors of the external environment and random disturbance term factors.

Infrastructure construction is the foundation of the economic development of a country. Research has been conducted from qualitative or quantitative perspectives to examine whether infrastructure construction has a catalytic effect on economic growth. Infrastructure development should be closely integrated with the local economic and social development level, to avoid unreasonable investments, unnecessary construction, excess capacity, and the waste of resources. Infrastructure development should consider the relationship between growth and distribution, efficiency, and equity; Aschauer [12] proposed, in 1989, that infrastructure investment significantly impacts economic growth and productivity improvement. Barro [13], in 1991, introduced the concept of public investment into the growth model, stating that infrastructure plays a long-term role in economic growth. Liao et al. [14], examining interprovincial panel data for China, showing that increased infrastructure investment has a general effect on sustained economic growth and that the growth effect has an “inverted U-shaped.” However, these studies focused on the impact of energy infrastructure on national economic indicators, ignoring its differential impact on different regions. In a study by Lin [15], in 2016, it was pointed out that to promote technological innovation and industrial upgrading, it is necessary to focus on increasing investments that can increase labor productivity or reduce costs, among which infrastructure is an important investment area. Yang [16], considering the current situation and future outlook for infrastructure investment, pointed out that, in terms of promoting continuous economic growth, urbanization will be the biggest driver in the following years and that infrastructure investment is a huge source of growth. The scale of new and traditional infrastructure investment will show a continuous growth trend. In the global energy forecast for 2021 [17], many institutions, including Bloomberg New Energy Finance (BNEF), unanimously pointed out that the energy sector would show investment and development in the global trend of promoting a low-carbon, environmentally friendly economy.

Infrastructure investment and its impact on the economy has been an object of long-standing research by scholars. Studies on the input–output efficiency of infrastructure in recent years have mainly focused on empirical evidence from cases, to prove whether infrastructure investment is conducive to promoting economic growth and narrowing the urban–rural gap. For example, Liu et al. [18], in 2010, conducted a regression on 21 years of panel data from 28 provinces and cities in China, to verify the impact of the construction and improvement of transportation infrastructure on promoting economic development. However, these studies did not address redistribution issues and rarely studied energy infrastructure investment. Energy is a pillar industry of the national economy, and energy infrastructure can affect economic growth from multiple perspectives. On the one hand, improving the efficiency of energy infrastructure investment can reduce the transfer costs of energy resources and promote the cross-regional accumulation and diffusion of production factors; on the other hand, improving the efficiency of energy infrastructure investment can effectively utilize local energy advantages and mitigate the impact of energy facility imbalance on economic development. Some studies further indicated that appropriate infrastructure investment strategies can narrow regional development gaps [19]. Another part of research on infrastructure investment and its impact on the economy points out that it may not contribute positively to the economy, but rather play a suppressive role. In a study by Zheng et al. [20], in 2012, it was demonstrated that infrastructure investment can have a crowding-out effect on human resources and inhibit the positive promotion of investment in the economy. Liao et al. [14], in their 2018 study, pointed out that infrastructure investment in 2012 and before positively affected the economy. However, after 2012, infrastructure investment did not function in forming effective social demand and, in turn, discouraged the entry of other investments besides those from the government to create a positive boost to economic development. The right strategy can effectively and equitably redistribute natural resources [21,22].

To summarize, among the studies on the investment efficiency of infrastructure and its impact on the regional development, there have been few targeted studies on the efficiency of energy infrastructure. Furthermore, the existing studies mainly focused on the impact of infrastructure investment on the overall indicators of the national economy, ignoring its differential impact on different regions. In addition, the reasons for the gradual change of the impact of infrastructure investment on the economy, from significant to insignificant, have been little explored in the existing literature. Furthermore, for electric power infrastructure investment, most domestic and foreign research has been conducted using one-stage DEA or SFA methods, and few studies have entered the third stage. This paper uses a unique investment project-based dataset of selected electricity power infrastructure investment projects in China’s Jiangsu Province as the research object, establishes an evaluation index system, evaluates their investment efficiency, and explores the impact of investment efficiency on the regional economy. Based on the empirical results, this paper further discusses the comprehensive impact of energy infrastructure construction on regional economic growth, stability, and industrial optimization, to obtain methods to improve the input–output efficiency of energy infrastructure and propose policies and development suggestions. The main structure of the full paper is organized as follows.

## 3. Analysis of the Current State of Energy Infrastructure Investment

### 3.1. Analysis of the Current Situation of Energy Infrastructure Investment in China

**Figure 1:**
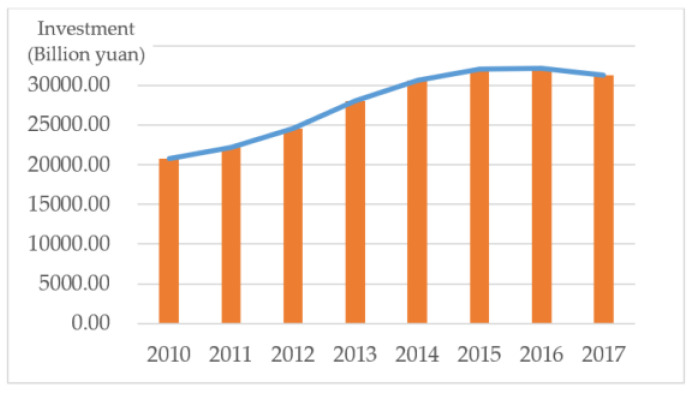
2010–2017 National Energy Investment Amount.

Focusing on energy infrastructure investment, this paper takes quantity of national energy investment as the main observable, to better explain China’s willingness toward energy investment. As seen in Figure 1, the amount of energy investment in China generally trended upward over the eight-year observation period from 2010–2017, peaking at 32,136 billion yuan in 2016, more than 1.5 times the amount invested in 2010.

In terms of the growth rate, as the base value becomes larger, the overall growth trend of China’s energy infrastructure investment gradually slows down. Due to the cyclical nature of the investment, there are certain changes from year to year, but most years maintain the growth trend, and with more than half of years having a growth rate of more than 5%, and the overall growth situation is more significant. 2012 to 2014 was a period of rapid growth for three consecutive years. The growth rate was maintained at about 10%, the growth rate gradually slowed down after 2014, and the first negative growth occurred in 2017, with a decline of 0.026%

**Figure 2:**
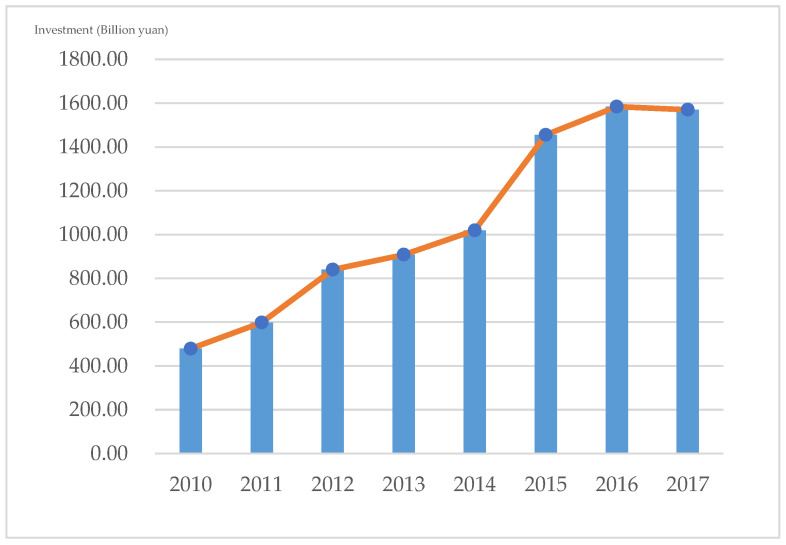
Amount of investment in the energy industry in Jiangsu Province, 2010–2017.

As shown in Figure 2, Jiangsu, one of the most important economic engines in China, and also as the main driver and pioneer of the energy transition, saw an overall increase in its amount of energy industry investment over the eight-year observation period from 2010 to 2017, again peaking at 1584 billion yuan in 2016.

At the regional level, as shown in Table 1, its share of the national energy investment has also been on the rise and has not yet reached its peak, predicting that Jiangsu Province will continue to maintain a steady increase in investment related to the energy industry. However, as the willingness to invest in energy infrastructure in Jiangsu province is limited by the heterogeneity of regional resource endowment and energy consumption structure, with the development of the economy, society, and technology, energy enterprises in each region must comply with the policy situation, strengthen their technology research and development, and make improvements in energy investment efficiency as a long-term strategic task, to build a complete and efficient energy supply system.

**Figure 3:**
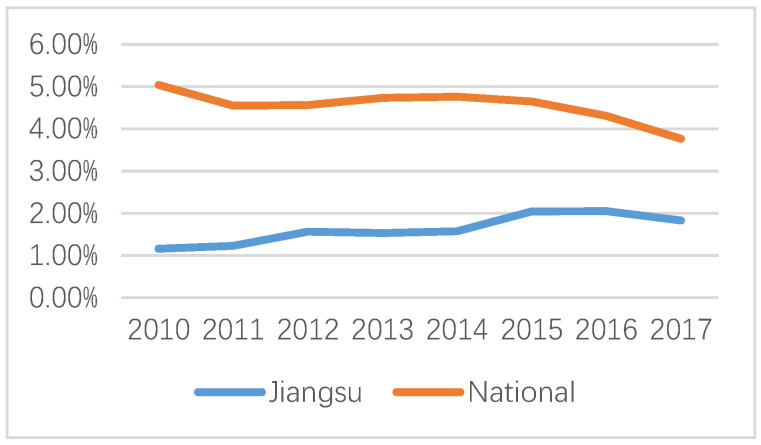
Change in the share of energy industry investment in GDP, 2010–2017.

As shown in Figure 3, national energy industry investment as a share of GDP showed a slow downward trend. The main reason for this was the decreasing dependence of the steadily climbing economic growth rate on the still increasing, but more moderately, energy demand. However, from a regional point of view, the share of energy industry investment in GDP in Jiangsu province rose slowly and then showed a downward trend. With rapid economic development, the industrial-based economy is gradually transforming into a service-based economy. Furthermore, technological progress and environmental awareness have driven an energy-saving transformation of energy. Various industries and residential life use electricity more widely, and energy use is more efficient. Hence, taking Jiangsu as a sample for analyzing the energy infrastructure issue, on one hand, it can serve as an effective contrast to the situation in developed countries. On the other hand, the presentation of the situation relating to the development of energy infrastructure in the best provinces of the Chinese economy can also serve as a benchmark for comparison with the development of other regions of China and other developing countries globally.

### 3.2. Analysis of Energy Infrastructure Investment Issues

The energy transition process has a complex impact on all sectors of the economy. For future development, energy is still in a major development period, under the goal of “net zero”. China’s energy and power industry, which used to be dominated by fossil fuels and resources, will gradually shift to a new direction; namely, renewable energy and clean energy. Under the guidance of the new energy security strategy of “four revolutions and one cooperation”, all aspects of the work of development, operation, emergency response, and technological innovation should continue to be carried out in depth.

In terms of the secure development of energy, it is necessary to continuously optimize the industrial structure layout of the electricity power infrastructure and build a strong security system. It is particularly important to strengthen the construction of supporting regional energy infrastructure, to provide security for industrial development. In China, it is necessary to consider the different advantages of energy in different regions, regulate the allocation and dispatch of resources, and promote the safety risk assessment of infrastructure construction, to ensure the safety and reliability of key facilities and equipment. In addition, energy infrastructure investment needs to further improve the program of emergency disposal, enhance the ability to regulate the response to extreme weather, and build an efficient and timely protection system for emergency disposal. We also need to promote the intelligent construction of energy infrastructure and the intelligent upgrading of the energy and electricity infrastructure construction chain.

The development of the economy and the improvement of the technological level provide a solid foundation for the development of new energy; how to rely on the economic foundation and technology accumulation, under the constraints of resource endowment, and continue to fight to develop energy infrastructure construction and improve its construction efficiency are undoubtedly urgent concerns in various industries.

Therefore, this study selects efficiency evaluation indexes of energy infrastructure construction investment, takes some electric power infrastructure investment projects in Jiangsu Province as the research object, evaluates the efficiency of their inputs and outputs, and tries to explore the impact of their investment efficiency on the regional economy, as well as putting forward policy suggestions to improve the efficiency of energy infrastructure project inputs and outputs and regional development based on empirical results and analysis.

## 4. Construction of Energy Infrastructure Investment Efficiency Evaluation Index System

### 4.1. Selection of Input–Output Indicators

When evaluating energy infrastructure investment efficiency based on the three-stage DEA model, establishing the input–output indicator system is crucial to whether valuable evaluation results can be obtained. Combining the characteristics of energy infrastructure investment and the availability of data, two types of indicators are used in this study when studying the efficiency of energy infrastructure investment: the first type is input indicators and output indicators; the second type is the external environment indicators. For the input indicators, actual monetary investment amounts were the variables been selected. For output indicator part, the capability for new infrastructures were the variables selected. The input–output indicator variables constructed in this paper are shown in Table 2.

### 4.2. Selection of Environmental Indicators

Environmental indicators are external influences, other than input–output indicators, that significantly impact the efficiency of energy infrastructure investment but are not within the control of the decision-making unit. In this paper, industrial structure IS (%), science and technology development level STL (thousand dollars), and openness to the outside world OUL (%) were selected as environmental variables (Table 3).

(1) Industrial structure

The share of the secondary industry in the regional economic structure was selected to represent the region’s industrial structure. In the regional development process, the industrial structure is an internal determinant of its industrial development, and energy infrastructure investment mainly involves the secondary industry. Different industrial structures will cause changes in the proportion of regional investment in different industries, the distribution of personnel, the output value, and other factors. Therefore, the industrial structure has an important impact on the efficiency of energy infrastructure investment.

(2) Science and technology level

The proportion of regional R&D expenditure to regional GDP was selected to represent the regional level of science and technology. The level of science and technology is an important component in the evaluation index of each region’s economic development level. An increase in R&D investment can promote the improvement of regional science and technology level and the construction level, affecting the efficiency of energy infrastructure investment.

(3) Degree of openness to the outside world

The proportion of import and export trade to the regional GDP expresses the indicator of the degree of external openness. In energy infrastructure investment project construction, an increase in regional market economy openness positively contributes to the efficiency of input and output. This is because a greater regional openness can attract more investment in the region, thus reducing the input of government-related sectors. It is also possible to learn and introduce advanced production technologies from the outside, which can represent a driving force and example in the region and further stimulate economic development.

## 5. Empirical Analysis of Energy Infrastructure Investment Efficiency Evaluation

### 5.1. Data Collection and Collation

#### 5.1.1. Data Collection

This study’s energy infrastructure investment data were obtained from the actual input and output data of 43 electric energy infrastructure construction projects in Jiangsu Province. The projects are spread across 13 cities in Jiangsu Province, with the largest number of projects being in Nantong (10) and the smallest number in Huai’an, Lianyungang, and Wuxi (1). Other projects, such as power supply and transmission, electric and railway power supply, new energy services, transmission channel strengthening, and network structure optimization framework all totaled six or fewer.

The environmental variables used in the SFA regression model in the second stage of this study were obtained from the Jiangsu Statistical Yearbook, the local statistical yearbooks and national economic and social development statistical bulletins of the 13 municipalities in Jiangsu Province, obtained from the China Economic and Social Big Data Platform of CNKI.

#### 5.1.2. Data Collation

In this study, the data of environmental variables collected corresponded to 43 projects according to project regions, to form a complete data system of project inputs, outputs, and environmental variables. Regarding the electricity infrastructure in China, it can usually be divided into four different categories: (1) Infrastructure to fit new electricity demand. (2) New energy related. (3) Transmission line reinforcement related. (4) Transmission network structure optimization. As the relationship between inputs and outputs varies among different project types, it is not appropriate to use the three-stage DEA model for uniform efficiency evaluation for all project. Since the data for infrastructure to fit new electricity demand was the most collected, this study focused on 19 infrastructure projects to meet new electricity demand for future evaluation. The statistics for the projects used can be found in Table 4.

In order to meet the requirements of the DEA model for the number of DMUs, i.e., decision-making units, in general, the number of decision-making units should be greater than or equal to the product of the number of input indicators and the number of output indicators, and not less than three-times the number of input indicators and output indicators [23], i.e., in this study, the number of DMUs needed to be greater than 15, so this paper only considered the projects for meeting demand for electricity class as the object of the investment efficiency evaluation. Among these 20 projects, only the A14 project in Suzhou had zero investment in line works, while the other projects had investment in substation works and line works. Therefore, to eliminate the impact of the zero investment in the A14 project on the efficiency evaluation, this study excluded the A14 project. After collation, this study identified 19 projects to meet electricity demand as the objects of investment efficiency evaluation. Descriptive statistics of the DMU inputs, outputs, and environmental variables are given in Table 4.

### 5.2. Empirical Evidence and Analysis of Results

#### 5.2.1. Phase 1: Raw Data DEA Efficiency Evaluation

In this study, MaxDEA was used to evaluate the investment efficiency of the original input and output data of 19 power infrastructure projects in Jiangsu Province from the meeting electricity demand category. The results are shown in Table 5. The last column of the efficiency evaluation results, RTS, indicates the payoff of scale, i.e., the level of the payoff of scale at which the current decision unit is located. Among these, increasing indicates increasing the payoff of scale, decreasing indicates decreasing the payoff of scale, and constant indicates a constant payoff of scale.

The evaluation of investment efficiency in the first stage showed that the average comprehensive technical efficiency of the 19 projects was 0.73, and the lowest comprehensive technical efficiency was 0.19; the average pure technical efficiency was 0.84, and the lowest was 0.47; the average scale efficiency was 0.85, and the lowest was 0.28. It can be seen that the comprehensive technical efficiency differences among projects were large, while the pure technical efficiency differences were small, and the comprehensive technical efficiency differences had a higher contribution to the scale efficiency differences. Furthermore, as shown in Table 6, the investment efficiency values were sorted by region, and it can be seen that the projects with efficiency 1, i.e., DEA effective, are concentrated in Yancheng, Zhenjiang, and Nantong, and the scale payoffs of all projects reached the constant scale payoff level. Among these, Yancheng had a total of five projects, and Zhenjiang had a total of one project, both of which had an investment efficiency of 1 and are located in the effective frontier, so in the first stage of the DEA efficiency evaluation, this study considered Yancheng and Zhenjiang as having the highest investment efficiency. Considering the number of projects and city development characteristics, the high investment efficiency of Yancheng and Zhenjiang may be due to the cluster effect of investment projects and the degree of industrial importance. Two of the five projects in Nantong had an investment efficiency of 1. The difference in the investment efficiency within the city was large, mainly due to the pure technical efficiency. It is worth noting that the comprehensive technical efficiency of Nanjing, Wuxi, Suzhou, and Yangzhou were all below the average, among which the pure technical efficiency and scale efficiency of Nanjing and Wuxi were both low, so this study concluded that Nanjing and Wuxi had the lowest project investment efficiency. This may be related to the economic development model and the cities’ economic development level.

#### 5.2.2. Phase 2: SFA Regression

The efficiency values obtained using the traditional data envelope model measured in the first stage may have been influenced by external environmental factors, random disturbance terms, and internal management inefficiencies, which can lead to errors in the measurement of energy infrastructure investment efficiency; this study used the SFA model for regression analysis, to remove the influence of environmental and luck factors on the efficiency evaluation. The implementation environment was the statistical software Frontier 4.1.

The explanatory variables in this stage of the SFA regression were the slack values of the input variables obtained in the first stage species, i.e., the amount of input reduction needed to reach DEA effectiveness. The explanatory variables were the three environmental variables of industrial structure, level of science and technology, and level of openness to the outside world, as described in the previous section. The regression can calculate the degree to which the environmental variables influence the slack values through parametric methods. According to the results of the SFA regression, the influence of the three dimensions of management inefficiency, external environmental factors, and random disturbance terms could be separated, so that the original inputs could be adjusted, i.e., the external environmental factors and random disturbance terms of all DMUs were adjusted to be the same, so that they were under the same external environment and level of luck. After removing the influence of these two dimensions, the new input variables were derived by regression prediction, and the results are shown in Table 7.

#### 5.2.3. Phase 3: DEA Efficiency Evaluation after Adjusting Inputs

After the adjustment of input indicators in the second stage, the efficiency evaluation of project inputs and outputs after excluding the influence of external environmental variables and random disturbance terms was again conducted in the third stage using the data envelope model, and the results are shown in Table 8. First, it can be seen by the average value that the integrated technical efficiency, pure technical efficiency, and scale efficiency all decreased, while the scale efficiency decreased relatively more, so this study considered that the adjusted efficiency decrease was mainly related to the correct measurement of scale efficiency.

After removing environmental variables and random disturbance terms, the efficiency values of each DMU become more compact and closer to the efficiency values, because of reducing differences such as socio-economic development level and luck factors. Further observation of the efficiency evaluation results sorted by region (Table 9) reveals that the scale efficiency of all projects in Nanjing, Suzhou, and Wuxi, as well as the original DEA effective projects in Nantong, were more than 0.1 compared to the first stage. The scale efficiency of all projects in Nanjing, Suzhou, and Wuxi, as well as the original DEA effective project in Nantong, decreased by more than 0.1 compared with the first stage. The scale payoff level was in the increasing stage. These four cities are all relatively economically developed cities in southern Jiangsu. This change indicates that the scale efficiency of urban electricity infrastructure investment in southern Jiangsu was insufficient when the projects of all cities were placed at a uniform level of socio-economic development, i.e., the high level of socio-economic development in southern Jiangsu has somewhat obscured the problem of the inefficiency of its electricity infrastructure investment. The main problem is that the scale of investment does not match the scale of socio-economic development. At the same time, the pure technical efficiency also has more room for progress compared to the many investment projects throughout Jiangsu province. This is supported by the positive changes in the two projects in Suqian City after adjusting their inputs.

On the other hand, four projects in Yancheng City and one project in Zhenjiang City were DEA-effective before and after adjusting the inputs, while there were DEA-effective projects in Nantong City. However, the proportion was small and fluctuated greatly after adjustment, and most of the projects turned out to be incremental in their scale payoff level, which may cause the problem of an insufficient investment scale. Therefore, this study concluded that Yancheng City and Zhenjiang City were the most efficient among the evaluated projects and regions in their power infrastructure investment, and where the relevant funds or resources were most fully utilized.

## 6. The Impact of Energy Infrastructure Investment on the Regional Economy

Energy infrastructure is a key component of China’s infrastructure construction and is important in promoting regional economic development. The efficiency of energy infrastructure investment has an important impact on all aspects of regional resource utilization, resource allocation optimization, and people’s living standards. However, in recent years, as China’s economy has entered a new normal, the role of energy infrastructure in promoting the regional economy has declined or even been inhibited, due to the upgrading of industries and transforming industrial structure. However, the downward pressure on the economy has increased due to the emergence of the new Covid-19 epidemic, the contraction of consumption, and the further increase of uncertainty in the international environment [24]. Infrastructure investment growth and efficiency will once again become an important part of stabilizing investment, improving the economy, promoting growth, and balancing regional development [25].

Therefore, it is especially important to pay attention to the efficiency of energy infrastructure investment, clarify its importance to the regional economy, and analyze the internal reasons for its impact. Improving the efficiency of energy infrastructure investment, giving full play to its positive role in promoting the healthy and effective development of the regional economy, is also of great significance to China’s social and economic development.

### 6.1. Indicator Selection and Data Sources

This study further explored the impact of the efficiency of energy infrastructure investment on the regional economy using the efficiency values of energy infrastructure investment derived from the previous three-stage DEA model as explanatory variables, regional economic development indicators as explanatory variables, and environmental factors that may have had an impact on the efficiency values affecting the regional economy as control variables. The indicators are specified as follows:

(1) Explanatory variables

In this chapter, the input–output efficiency values of energy infrastructure construction projects after adjusting the values of input indicator variables in the third stage of the three-stage DEA regression results in Section 5 were selected as the explanatory variables to represent the regional investment efficiency level and further explore its impact on the regional economy.

(2) Control variables

The environmental factors that may have impacted the mechanism of efficiency values affecting the regional economy, the level of science and technology, the degree of openness to the outside world, and the industrial structure were selected as control variables in this study, and these variables are shown in Table 3. Since the level of science and technology in a region largely determines the level of regional productivity, it plays a key role in developing a regional economy. It helps to promote the increase in the utilization of production factors. In addition, the degree of openness to the outside world determines the degree of freedom of the regional economy and the ability to attract foreign investment, which is helpful for the introduction of advanced technology and high-level talents from outside the region, etc., and is especially important for regional economic development. The development of the regional economy is closely related to the change, upgrade, and optimization of the industrial structure of each region, and the emergence of new industries promotes the transformation of the regional economy towards a high quality level and helps to improve the efficiency of regional labor production. Therefore, in this study, the level of science and technology STL was determined by the ratio of regional R&D expenditure investment to regional GDP, the degree of openness to the outside world OUL was determined by the ratio of regional total import and export trade to regional GDP, and the industrial structure IS was determined using the ratio of regional secondary industry to regional economic structure.

(3) Explained variables

For the indicators of regional economic measurement, this study considered, not only the level and speed of economic development, but also whether the economic development met the requirements of a high level, high quality, and sustainable and stable development. Jin [26] pointed out in his study on economic development that the criteria for judging the level and quality of economic development are whether economic development can meet the growing needs of the people for a better life. Other scholars, such as Li et al., suggested the need to pay attention to the impact of economic structural changes [27], as well as pointing out that the structure of regional industries, the structure of residents’ consumption, and the structure of urban and rural areas are all important parts of regional economic development.

Therefore, in studying the regional economy, this study constructed an index system for measuring the regional economy from three perspectives: the speed of economic growth, the stability of economic growth, and the optimization of the industrial structure in economic growth. For the speed of economic growth, the GDP per resident and the region’s GDP growth rate were secondary indicators. For the stability of economic growth, the urban residents’ unemployment rate and the residents’ price consumption index were selected as secondary indicators. For the optimization of industrial structure, the industrial structure transfer ratio and the gross industrial product were selected as secondary indicators. The ratio of the number of registered unemployed persons to the total population at the end of the year determined the unemployment rate of urban residents. The industrial structure transfer ratio was determined from the added value of secondary and tertiary industries in the regional economic structure; the data of the remaining indicators were directly obtained from the National Bureau of Statistics, China Economic and Social Data Research Platform, Jiangsu Statistical Yearbook, etc. Describe for indicators could be found in Table 10.

### 6.2. Empirical Evidence and Analysis of Results

#### 6.2.1. Entropy Method Index System

From the perspectives of subjectivity and objectivity, index system assignment methods include the following two categories. Subjective scoring, composite index, Delphi, etc., are more mature subjective assignment methods. Although these methods have convenient features, they are controversial due to their high subjectivity. The other type is to assign weights to each index from an objective point of view, including factor analysis methods, principal component analysis methods, entropy methods, etc. [28]. The weighting of indicators from an objective perspective has the characteristics of strong objectivity and high rationality. In this study, the entropy value method was used to assign weights to the index system, and the steps were as follows:

(1) Data standardization

Due to the different units and orders of magnitude of the original data, they were standardized in this study, to facilitate comparison and evaluation. In addition, in the selected indicator system, some indicators are positive, i.e., the larger the value of the indicator variable, the more obvious the impact. In contrast, some indicators are negative, i.e., the smaller the value of the indicator variable, the more significant the effect. Therefore, this study utilized the indicator magnitude, units, and attributes. (*i* in the formula denotes the decision unit and *j* denotes the indicator)

Positive indicators.

(1) $$X_{ij}^{\prime }=\frac{X_{ij}-\min\left(X_{j}\right)}{\max\left(X_{j}\right)-\min\left(X_{j}\right)}$$

Negative indicators.

(2) $$X_{ij}^{\prime }=\frac{\max\left(X_{j}\right)-X_{ij}}{\max\left(X_{j}\right)-\min\left(X_{j}\right)}$$

(2) Calculation of the weight of the index value

(3) $$y_{ij}=\frac{X_{ij}^{\prime }}{\sum_{i}X_{ij}^{\prime }}$$

(3) Calculate the information entropy of indicators

(4) $$e_{j}=-k\sum_{i}y_{ij}\times \ln y_{ij}\;$$

where *k* is the normal number and let $k=\frac{1}{ln\;m}$, *m* be the number of samples, the k>0, and $0\leq e_{i}\leq 1$, when $y_{i}=0$ when $y_{i}\ln y_{i}=0$

(4) Calculate the coefficient of variation of indicators

(5) $$d_{j}=1-e_{j}$$

(5) Calculate the weights of indicators

(6) $$w_{j}=\frac{d_{j}}{\sum^{\;}d_{j}}$$

(6) Calculate the score of the index

(7) $$u_{i}=\sum_{j}y_{ij}w_{j}$$

After the above steps of the index system using the entropy value method, the weight of the indexes was obtained, as shown in Table 11.

#### 6.2.2. Empirical Regression and Analysis of Results

After using the above indicator system with variables and regression using Stata15.1 software, the results are shown in Table 12. The results in the table show that in the regression of the impact of energy infrastructure investment efficiency on the regional economy, without adding control variables, the result is that the two are significantly negatively correlated, at a 99.9% confidence level. In addition, since the result is for cross-sectional data, the result is valid for observations. In the case of adding only a single control variable, the coefficient of the key indicator, i.e., the infrastructure investment efficiency, remains significantly negative, and the significance decreases only when the STL variable is added as a control variable, i.e., the STL variable is not significant. With the inclusion of two control variables, the key indicator is significantly negatively correlated at a 99.9% confidence level only when the OUL and IS indicators are included, where the IS variable is insignificant. Including three control variables, the key indicators are not significant. From the regression results, it can be concluded that the control effect of adding two control variables versus three control variables is not significant, so in the following, we focus on the case of adding one control variable.

Based on the regression results and concerning the actual development of the region, this study interprets the regression results as follows: Overall, the regression coefficient of the infrastructure investment efficiency index was negative, indicating that the driving effect of energy infrastructure on the quality of regional economic development was not reflected. This may be because the energy infrastructure in Jiangsu Province had been of a certain scale for a few decades, after which the annual investment became mainly focused on facility maintenance and local construction and repair, not a process from nothing to something. Further analysis shows that since the economic development of southern Jiangsu has reached a high level, the investment in energy infrastructure is in the stage of filling the gap, so the driving effect on regional economic growth was not obvious, and even the marginal return decreased. Moreover, according to the regression results of the breakdown of the efficiency values on the indicators of industrial structure optimization, it can be concluded that the efficiency of energy infrastructure investment made a significant positive contribution to the process of regional transformation from a natural economy to urban socialized mass production and to the increase in industrial structure level, which was due to the fact that the increase in the efficiency of investment in energy infrastructure construction caused an increase in the public infrastructure industrial input, which formed the industrial production capacity, and this further caused an increase in its own output created, which increased the social wealth, promoted the transformation of the industrial structure to secondary and tertiary industries, and facilitated the transformation of the region from a natural economy to urban socialized mass production.

Thus, it can be seen that the improvement of energy infrastructure investment efficiency had a key role in increasing the social wealth and transforming the regional industrial structure and economy. However, it was not reflected in the level and quality of regional economic development. In addition, the improvement of energy infrastructure investment efficiency contributed to the improvement of the energy industry structure, the improvement of resource utilization efficiency, the improvement of industrial production and residents’ living conditions, and increased the stability of resource safety and environmental security. Therefore, improving the efficiency of investment in energy infrastructure construction is important, to allow high-quality economic development and promote better local environmental conditions.

## 7. Conclusions and Recommendations

### 7.1. Conclusions

This study focused on energy infrastructure investment, constructed an energy infrastructure investment efficiency evaluation system and a three-stage DEA efficiency evaluation model, further studied its impact on regional development, and drew the following main conclusions:

(1) In the DEA efficiency evaluation, in each region, the comprehensive technical efficiency differences among energy infrastructure construction projects were large. In contrast, the pure technical efficiency differences were small, and the contribution of scale efficiency differences to the comprehensive technical efficiency differences was high.
(2) In the third stage of DEA efficiency evaluation, after adjusting the inputs and when the projects of all cities were placed at a uniform socio-economic development level, the scale efficiency of urban electricity infrastructure investment in southern Jiangsu was insufficient and the socio-economic development level blurred its electricity infrastructure investment inefficiency to some extent.
(3) By analyzing the regression results, this study concludes that the improvement of the efficiency of energy infrastructure investment at the level of regional economic development was not reflected. However, it had a key role in increasing social wealth and transforming the regional industrial structure and economy.

### 7.2. Recommendations

The results of this paper show that the efficiency of various energy infrastructure investment and construction projects in Jiangsu province was uneven and presented a wide range of characteristics, which were highly correlated with the economic development pattern and industrial structure of each city. These scenarios occur in other regions of China and in other developing countries. Considering the impact of energy infrastructure on the regional economy and its important role in increasing social wealth and upgrading the industrial structure, it is particularly important to propose reasonable and effective recommendations to improve the efficiency of energy infrastructure investments. In order to maximize the efficiency of energy infrastructure investment, improve infrastructure investment management, and strive to achieve the optimal investment scale, this study gives the following specific recommendations, by combining the empirical results and the actual situation and social operation related to economic development and environmental protection.

(1) Planning a reasonable scale of investment

Planning a reasonable scale of investment is important, to determine the most effective input under the premise of ensuring the maximum scale of output. For energy infrastructure investment, a certain scale of output must be achieved, in order to generate benefits. However, when making investment decisions, one needs to consider the stage the region is currently in. Whether energy infrastructure investment is at a stage where demand is not being met or is in a state of leak detection is a vital consideration before planning is undertaken. In addition, energy infrastructure investment needs to be measured in a holistic manner. In a situation where infrastructure investment efficiency shows diminishing returns to scale, it is important to rationalize its remaining capacity and optimize the layout of energy infrastructure, by opening construction and production between different regions, to improve efficiency. Therefore, the search for the best and most reasonable scale of investment should consider the existing infrastructure stock and include the stock of adjacent regions, to maximize the scale benefits of energy infrastructure investment as much as possible.

(2) Implementation of differentiated regional investment

The differences in economic development and resource distribution among regions lead to significant differences in the efficiency of energy infrastructure investments. Therefore, it is particularly important to promote inter-regional coordination and balance. The scale of investment in each region should be adapted to the actual situation of the regional economy, and investment decisions should follow local economic development and electricity consumption conditions. Cities with high levels of economic development should pay attention to optimizing regional investment, to avoid waste of resources and loss of efficiency due to duplicated construction and over-investment. Resource-based cities and cities with a slightly lower level of economic development should continue to take advantage of their own strengths, maintain a high level of energy infrastructure investment efficiency, make full use of their advantages to improve the utilization rate of resources, and optimize their investment structure to promote their energy infrastructure investment efficiency.

(3) Enhancement of management and technology level

The investment decision-making ability of each region’s energy infrastructure has an important impact on its investment efficiency. Relevant departments and power companies should improve their management level and employ excellent management and construction talents, to ensure the effectiveness and scientific basis of decision-making. In addition, the analysis found that a large part of the inefficiency of energy infrastructure investment was due to its failure to achieve technological effectiveness with constant returns to scale. Therefore, in order to raise the level of technology and improve technical efficiency, government departments need to introduce corresponding policies, to stimulate technological innovation and technology upgrading, allocate resources rationally, and avoid wasting resources.

## Figures and Tables

**Table 1:** Jiangsu’s share of national energy investment, 2010–2017.

	2010	2011	2012	2013	2014	2015	2016	2017
Jiangsu’s proportion of national energy investment	2.31%	2.69%	3.42%	3.24%	3.33%	4.54%	4.93%	5.01%

**Table 2:** Selection of input–output indicators.

	Variable Name	Variable Units	Variable Description
Inputs Indicators	SDI for dynamic investment in substation projects	million yuan	At the time of completion and settlement, the dynamic investment amount of the substation project (including tax)
	Line engineering dynamic investment LDI	million yuan	Dynamic investment amount of line works (including tax) at the time of completion and settlement
Outputs Indicators	New substation capacity NFC	MVA	The new value-added capacity of the main transformer of the power grid substation
	New line length NLL	Kilometers	The new length of transmission line construction
	New transmission capacity NMC	MW	The maximum transmission system power allowed to be delivered between power systems

**Table 3:** Selection of environmental variables.

	Variable Name	Variable Units	Variable Description
Environment Indicators	Industrial Structure IS	%	The share of the secondary sector in the regional economic structure
	Science and Technology Level STL	%	Regional R&D investment as a percentage of regional GDP
	Degree of openness to the outside world OUL	%	Total regional import and export trade as a percentage of regional GDP

**Table 4:** Descriptive statistics of DMU inputs, outputs, and environmental variables.

Variable	Obs	Mean	Std. Dev.	Min	Max
SDI	19	12,797.13	8490.869	2953.31	34,200.18
LDI	19	8913.738	8458.116	1591.05	33,761.8
NFC	19	757	807.1086	63	2000
NLL	19	44.349	37.16413	3.6	141.44
NMC	19	3881.825	5116.319	0	13856
IS	19	44.61053	4.463916	35.9	49
STL	19	0.002632	0.006534	0	0.02
OUL	19	0.312632	0.313561	0.08	1.14

**Table 5:** Investment efficiency values for the first phase of the 19 projects from the meeting the electricity demand category (sorted by DMU).

DMU	Local Municipalities	TE	PTE	SE	RTS
A01	Suzhou	0.81	0.85	0.95	Increasing
A02	Suzhou	0.58	0.61	0.96	Increasing
A05	Yancheng	1	1	1	Constant
A06	Xuzhou	0.85	0.96	0.88	Decreasing
A08	Yancheng	1	1	1	Constant
A11	Nantong	1	1	1	Constant
A12	Zhenjiang	1	1	1	Constant
A15	Yangzhou	0.59	1	0.59	Decreasing
A16	Yancheng	1	1	1	Constant
A17	Suqian	0.69	0.84	0.83	Decreasing
A20	Nanjing	0.31	0.47	0.67	Increasing
A29	Nantong	0.52	0.52	0.99	Increasing
A31	Suqian	0.60	0.62	0.95	Decreasing
A32	Nantong	0.69	0.78	0.89	Decreasing
A34	Nantong	0.80	0.84	0.94	Decreasing
A35	Yancheng	1	1	1	Constant
A38	Nanjing	0.19	0.69	0.28	Increasing
A40	Wuxi	0.26	0.76	0.34	Increasing
A41	Nantong	1	1	1	Constant
Average value		0.73	0.84	0.85	

**Table 6:** Investment efficiency values of the first phase of the 19 projects to meet the demand for electricity (sorted by the municipality).

DMU	Local Municipalities	TE	PTE	SE	RTS (Remuneration for Scale)
A20	Nanjing	0.31	0.47	0.67	Increasing
A38	Nanjing	0.19	0.69	0.28	Increasing
A11	Nantong	1	1	1	Constant
A29	Nantong	0.52	0.52	0.99	Increasing
A32	Nantong	0.69	0.78	0.89	Decreasing
A34	Nantong	0.8	0.84	0.94	Decreasing
A41	Nantong	1	1	1	Constant
A01	Suzhou	0.81	0.85	0.95	Increasing
A02	Suzhou	0.58	0.61	0.96	Increasing
A17	Suqian	0.69	0.84	0.83	Decreasing
A31	Suqian	0.6	0.62	0.95	Decreasing
A40	Wuxi	0.26	0.76	0.34	Increasing
A06	Xuzhou	0.85	0.96	0.88	Decreasing
A05	Yancheng	1	1	1	Constant
A08	Yancheng	1	1	1	Constant
A16	Yancheng	1	1	1	Constant
A35	Yancheng	1	1	1	Constant
A15	Yangzhou	0.59	1	0.59	Decreasing
A12	Zhenjiang	1	1	1	Constant
Average value		0.73	0.84	0.85	

**Table 7:** The 19 projects to meet the electricity demand and the phase input adjustment (sorted by DMU).

DMU	Local Municipalities	Value before Input 1 Adjustment	Input 1 Adjusted Value	Value before Input 2 Adjustment	Input 2 Adjusted Value
A01	Suzhou	3872.95	6224.05	3669.93	10,428.35
A02	Suzhou	10,779.8	16,746.21	11,286.4	18,044.82
A05	Yancheng	16,987.14	18,781.92	4798.44	4798.44
A06	Xuzhou	21,870.54	244,31.52	24,541.42	24,982.70
A08	Yancheng	20,895.71	22,690.49	1591.05	1591.05
A11	Nantong	25,255.04	27,058.14	3998.49	6763.95
A12	Zhenjiang	18,037.17	19,839.17	2869.61	5199.75
A15	Yangzhou	34,200.18	36,001.40	33761.8	35,726.92
A16	Yancheng	18,392.89	20,187.67	15,499.24	15,499.24
A17	Suqian	6642.21	10,818.82	3331.47	4206.06
A20	Nanjing	11,842.14	19,812.48	11,824.46	20,244.65
A29	Nantong	9119.49	15,221.24	8454.36	11,219.82
A31	Suqian	6459.34	10,640.82	4395.64	5270.23
A32	Nantong	8275.18	12,504.26	7183.69	9949.15
A34	Nantong	10,091	13,973.48	10,191.46	12,956.92
A35	Yancheng	8815.05	10,609.83	14,395.46	14,395.46
A38	Nanjing	4286.41	7429.73	3194.7	11,614.89
A40	Wuxi	4369.95	7220.75	2481.01	8462.54
A41	Nantong	2953.31	4756.41	1892.39	4657.85

**Table 8:** Phase III investment efficiency values for the 19 projects for meeting electricity demand (sorted by DMU).

DMU	Local Municipalities	TE	PTE	SE	RTS
A01	Suzhou	0.53	0.82	0.65	Increasing
A02	Suzhou	0.42	0.50	0.84	Increasing
A05	Yancheng	1	1	1	Constant
A06	Xuzhou	0.84	0.93	0.90	Decreasing
A08	Yancheng	1	1	1	Constant
A11	Nantong	1	1	1	Constant
A12	Zhenjiang	1	1	1	Constant
A15	Yangzhou	0.63	1	0.63	Decreasing
A16	Yancheng	1	1	1	Constant
A17	Suqian	1	1	1	Constant
A20	Nanjing	0.20	0.37	0.55	Increasing
A29	Nantong	0.48	0.53	0.89	Increasing
A31	Suqian	0.79	0.85	0.92	Increasing
A32	Nantong	0.67	0.71	0.93	Increasing
A34	Nantong	0.71	0.73	0.96	Increasing
A35	Yancheng	1	1	1	Constant
A38	Nanjing	0.11	0.64	0.18	Increasing
A40	Wuxi	0.15	0.67	0.23	Increasing
A41	Nantong	0.75	1	0.75	Increasing
Average value		0.70	0.83	0.81	

**Table 9:** The 19 projects to meet electricity demand, the third stage investment efficiency values (sorted by local municipalities).

DMU	Local Municipalities	TE	PTE	SE	RTS
A20	Nanjing	0.2	0.37	0.55	Increasing
A38	Nanjing	0.11	0.64	0.18	Increasing
A11	Nantong	1	1	1	Constant
A29	Nantong	0.48	0.53	0.89	Increasing
A32	Nantong	0.67	0.71	0.93	Increasing
A34	Nantong	0.71	0.73	0.96	Increasing
A41	Nantong	0.75	1	0.75	Increasing
A01	Suzhou	0.53	0.82	0.65	Increasing
A02	Suzhou	0.42	0.5	0.84	Increasing
A17	Suqian	1	1	1	Constant
A31	Suqian	0.79	0.85	0.92	Increasing
A40	Wuxi	0.15	0.67	0.23	Increasing
A06	Xuzhou	0.84	0.93	0.9	Decreasing
A05	Yancheng	1	1	1	Constant
A08	Yancheng	1	1	1	Constant
A16	Yancheng	1	1	1	Constant
A35	Yancheng	1	1	1	Constant
A15	Yangzhou	0.63	1	0.63	Decreasing
A12	Zhenjiang	1	1	1	Constant
Average value		0.70	0.83	0.81	

**Table 10:** System of indicators for measuring the regional economy.

Tier 1 Indicators	Secondary Indicators	Indicator Unit	Indicator Description
Economic Growth	GDP per capita	Yuan	/
	GDP growth rate	%	/
Economic Stability	The unemployment rate of urban residents	%	The ratio of the number of registered unemployed persons in urban areas at the end of the year to the total population at the end of the year
	Consumer Price Index	%	/
Industry Optimization	Industrial structure transfer ratio	%	Value added of secondary and tertiary industries as a proportion of the regional economic structure
	Gross Industrial Product	Yuan	/

**Table 11:** Index weights calculated using the entropy value method.

Tier 1 Indicators	Secondary Indicators	Indicator Weighting
Economic Growth	GDP per capita	0.1748
	GDP growth rate	0.213
Economic Stability	The unemployment rate of urban residents	0.1346
	Consumer Price Index	0.2497
Industry Optimization	Industrial structure transfer ratio	0.0638
	Gross Industrial Product	0.1641

**Table 12:** Empirical regression results.

	(1)	(2)	(3)	(4)	(5)	(6)	(7)	(8)
	LE	LE	LE	LE	LE	LE	LE	LE
TE	−0.0523 ***	−0.0624 **	−0.0380 ***	−0.0551 ***	−0.0358	−0.0329	−0.0435 ***	−0.0114
	(−5.36)	(−3.77)	(−4.71)	(−6.80)	(−2.12)	(−2.08)	(−4.69)	(−0.78)
STL		−0.666			0.115	1.619		2.135 **
		(−1.13)			(0.22)	(2.11)		(3.23)
OUL			0.0268 ***		0.0276 ***		0.0200 **	0.0248 **
			(6.17)		(4.08)		(3.30)	(3.66)
IS				0.00152 *		0.00272 ***	0.00103	0.00250 ***
				(2.81)		(4.60)	(1.83)	(4.36)
_cons	0.0894 ***	0.0993 ***	0.0709 ***	0.0237	0.0687 ***	−0.0526	0.0310	−0.0678
	(13.40)	(7.23)	(13.20)	(1.16)	(4.55)	(−1.47)	(1.51)	(−1.99)
*N*	19	19	19	19	19	19	19	19

*t* statistics in parentheses. * *p <* 0.05, ** *p* < 0.01, *** *p* < 0.001.

## Data Availability

The data presented in this study are available on request from the corresponding author.
